# Effectiveness and cost-effectiveness of Improving clinicians’ diagnostic and communication Skills on Antibiotic prescribing Appropriateness in patients with acute Cough in primary care in CATalonia (the ISAAC-CAT study): study protocol for a cluster randomised controlled trial

**DOI:** 10.1186/s13063-019-3727-3

**Published:** 2019-12-17

**Authors:** Rafa Ruiz, Ana Moragas, Marta Trapero-Bertran, Antoni Sisó, Anna Berenguera, Glòria Oliva, Alícia Borràs-Santos, Ana García-Sangenís, Jaume Puig-Junoy, Josep M. Cots, Rosa Morros, Toni Mora, Anna Lanau-Roig, Ramon Monfà, Amelia Troncoso, Rosa M. Abellana, Pau Gálvez, Laura Medina-Perucha, Lars Bjerrum, Isabel Amo, Nieves Barragán, Carl Llor

**Affiliations:** 1 Institut Català de la Salut, Barcelona, Spain; 2 Fundació Institut Universitari per a la recerca a l’Atenció Primària de Salut Jordi Gol i Gurina (IDIAPJGol), Barcelona, Spain; 3 Universitat Rovira i Virgili, Jaume I Health Centre, Institut Català de la Salut, Tarragona, Spain; 4 Research Institute for Evaluation and Public Policies (IRAPP), Universitat Internacional de Catalunya, Barcelona, Spain; 5 Fundació Atenció Primària, Barcelona, Spain; 6 Fundació Institut Universitari per a la recerca a l’Atenció Primària de Salut Jordi Gol i Gurina (IDIAPJGol), Universitat Autònoma de Barcelona, Bellaterra, Cerdanyola del Vallès, Spain; 7 Ministry of Health, Government of Catalonia, Barcelona, Spain; 8 Institut Universitari de Pacients (Patients’ University Institut), Universitat Internacional de Catalunya, Barcelona, Spain; 9 Pompeu Fabra University (UPF)-Barcelona School of Management, Barcelona, Spain of Economics and Business, Barcelona, Spain; 10 Universitat de Barcelona, La Marina Health Centre, Institut Català de la Salut, Barcelona, Spain; 11 La Marina Health Centre, Institut Català de la Salut, Associació d’Infermeria Familiar i Comunitària de Catalunya, Barcelona, Spain; 12 Fundació Institut Universitari per a la recerca a l’Atenció Primària de Salut Jordi Gol i Gurina (IDIAPJGol), UICEC de IDIAP Jordi Gol – Plataforma SCReN, Universitat Autònoma de Barcelona, Bellaterra, Cerdanyola del Vallès, Spain; 13 Àrea de Suport al Medicament i Servei de Farmàcia Barcelona, Institut Català de la Salut, Barcelona, Spain; 14 Biostatistics, Department of Basic Clinical Practice, Universitat de Barcelona, Barcelona, Spain; 15 Centre for General Practice, Department of Public Health, University of Copenhagen, Copenhagen, Denmark; 16 Catalan Society of Family Medicine, Group on Communication, Health Centre Vallcarca, Barcelona, Spain; 17 Fundació Institut Universitari per a la recerca a l’Atenció Primària de Salut Jordi Gol i Gurina (IDIAPJGol), Manso Health Centre, Institut Català de la Salut, Barcelona, Spain

**Keywords:** Cost-effectiveness, Cost-utility, Effectiveness, Anti-bacterial agents, Prescribing, Acute cough, Respiratory tract infections, Antimicrobial stewardship, Qualitative research, Incremental cost-utility ratio, Primary healthcare

## Abstract

**Background:**

Despite their marginal benefit, about 60% of acute lower respiratory tract infections (ALRTIs) are currently treated with antibiotics in Catalonia. This study aims to evaluate the effectiveness and efficiency of a continuous disease-focused intervention (C-reactive protein [CRP]) and an illness-focused intervention (enhancement of communication skills to optimise doctor-patient consultations) on antibiotic prescribing in patients with ALRTIs in Catalan primary care centres.

**Methods/design:**

A cluster randomised, factorial, controlled trial aimed at including 20 primary care centres (*N* = 2940 patients) with patients older than 18 years of age presenting for a first consultation with an ALRTI will be included in the study. Primary care centres will be identified on the basis of socioeconomic data and antibiotic consumption. Centres will be randomly assigned according to hierarchical clustering to any of four trial arms: usual care, CRP testing, enhanced communication skills backed up with patient leaflets, or combined interventions. A cost-effectiveness and cost-utility analysis will be performed from the societal and national healthcare system perspectives, and the time horizon of the analysis will be 1 year. Two qualitative studies (pre- and post-clinical trial) aimed to identify the expectations and concerns of patients with ALRTIs and the barriers and facilitators of each intervention arm will be run. Family doctors and nurses assigned to the interventions will participate in a 2-h training workshop before the inception of the trial and will receive a monthly intervention-tailored training module during the year of the trial period. Primary outcomes will be antibiotic use within the first 6 weeks, duration of moderate to severe cough, and the quality-adjusted life-years. Secondary outcomes will be duration of illness and severity of cough measured using a symptom diary, healthcare re-consultations, hospital admissions, and complications. Healthcare costs will be considered and expressed in 2021 euros (year foreseen to finalise the study) of the current year of the analysis. Univariate and multivariate sensitivity analyses will be carried out.

**Discussion:**

The ISAAC-CAT project will contribute to evaluate the effectiveness and efficiency of different strategies for more appropriate antibiotic prescribing that are currently out of the scope of the actual clinical guidelines.

**Trial registration:**

ClinicalTrials.gov, NCT03931577.

## Background

Overuse of antibiotics has contributed to the development of antimicrobial resistance, which represents a major threat to human health worldwide [1]. In addition to driving resistance, non-indicated use of antibiotics incurs costs, increases re-consultation for subsequent episodes, medicalizes self-limiting illness, and unnecessarily exposes individuals to the risk of side effects. Most antibiotics are prescribed in primary care, most commonly for acute lower respiratory tract infections (ALRTI), which account for one-fourth of all the infectious diseases treated by general practitioners (GPs). According to a study carried out in Catalonia, about 60% of ALRTIs are currently treated with antibiotics [2]. The latest Cochrane review also suggests that only a slight benefit is achieved from the prescription of antibiotics [3]. Because no new classes of antibiotics have been developed in the last two decades, rationalisation of antibiotic use in the treatment of ALRTIs in primary care is a key target for influencing the antibiotic-prescribing behaviour of clinicians [1]. This supports the case for strategies that promote a more prudent use of antibiotics for these infections. This is even more important in Catalonia, because this is one of the European Union countries with the highest rates of antibiotic prescribing, which has even slightly increased in the last years [4, 5]. This finding is also supported by the results of the latest Eurobarometer on antibiotic use carried out in 2016, in which 47% of the Spanish respondents admitted having taken an antibiotic in the previous year [6].

Antibiotic use can be improved by using educational interventions such as dissemination of printed and audio-visual educational materials, group education, personal or group feedback, individual outreach visits, reminders at the time of prescribing, and computer-assisted decision-making systems, among others, but these interventions generally result in little change in prescription practices, although the effect is greater with multi-faceted approaches [7, 8]. In a recent Cochrane review of systematic reviews aimed at assessing the effects of interventions targeting clinician antibiotic prescribing for ALRTIs in primary care, the reviewers found that the most powerful tools for reducing unnecessary antibiotic prescribing are C-reactive protein (CRP) rapid testing, shared decision making, and procalcitonin-guided management without compromising outcomes of patient satisfaction and re-consultation, although the measurement of these outcomes was limited in the trials [9].

The antibiotic prescribing behaviour of GPs for acute cough in adults is influenced by their perceptions of patient expectations, but sometimes their perceptions do not match patient views [10]. Patients report lower levels of satisfaction with their consultation if they have an expectation for antibiotics that is not met, and being able to effectively elicit patient views towards shared decisions is therefore an important skill for clinicians managing acute cough. Interactive workshops for healthcare professionals and education of patients are likely to lower the rate of antibiotic prescribing [10]. A Dutch study found that the training of physicians in advanced communication skills by seminar role-playing and peer feedback on consultation transcripts reduced antibiotic prescribing rates by 20% [11]. In a Welsh study a 4% reduction in global antibiotic use was found after web-based training in advanced communication skills [12].

CRP testing has been shown to safely reduce antibiotic prescribing in primary care [13]. In a Dutch study, training of physicians in CRP testing lowered the rate of antibiotic prescribing by 20%, but the greatest reduction was observed among GPs assigned to CRP testing and enhanced communication skills training [11]. In a multinational study conducted in Europe, which assessed the effects of internet-based training on antibiotic prescribing for acute respiratory tract infections, both CRP and clinician training in communication skills were effective in reducing antibiotic prescribing, but the greatest reduction was also seen when both the CRP testing and communication skills training interventions were combined [14]. The effect of the communication skills training was greater in the Dutch study, which could be explained mainly by the fact that the intervention was more intense and continuous. In the latter study, however, the intervention was limited to a single online training lasting approximately 1 h before the trial started. In addition, patients were followed for only 4 weeks, whereas approximately 25% of patients still have cough after this period, and patients still re-attended their GPs because full recovery was not accomplished.

Recent evidence showed that ambulatory antibiotic prescription is associated with a hidden cost of antibiotic resistance that substantially increases the cost of an antibiotic prescription [15]. This finding raises concerns regarding the magnitude of misalignment between individual and societal antibiotic costs. There is a lack of evidence evaluating the efficiency of different multi-faceted approaches in order to decrease the inappropriate prescribing of antibiotics in Mediterranean countries. Although CRP point-of-care tests are widely used in a number of European countries for the management of ALRTIs, they are not yet routinely used in Catalonia. In addition, these overprescribing actions could lead to inefficiencies of the health system.

## Methods/design

### Objectives

This study aims to evaluate the effectiveness and efficiency of a continuous (workshop and monthly web-based training) disease-focused intervention (CRP testing) and a continuous (on-site and monthly online training) illness-focused intervention (enhancement of communication skills to optimise doctor-patient consultations and share decision making with the aid of patient-centred leaflets) on antibiotic prescribing in patients with ALRTIs in Catalan primary care centres. An economic evaluation of four strategies for reducing antibiotic prescribing will be performed. Particularly, a cost-effectiveness and cost-utility analysis will be conducted. These interventions will be compared with the current antibiotic prescribing strategy used in the primary care centres. The analysis will be conducted from the societal and the national healthcare system perspectives. Two qualitative studies (pre- and post-clinical trial) will be also conducted to inform the development and evaluation of the clinical trial.

### Study design

A cluster randomised, factorial, controlled trial performed over a period of 18 months including two autumn-winter seasons will be carried out. A randomised cluster design will be used to keep contamination (influence on participants’ behaviour when another participant or physician alters his/her behaviour) within primary care centres to a minimum, because three GPs and nurses per centre are expected to participate and because a centre-based meeting is part of the intervention. Patients who fulfil the inclusion criteria will be given written and verbal information about the study and will be asked to provide written informed consent.

The inclusion criteria will be age older than 18 years and first consultation for acute cough (new cough or worsening of a previous cough) of up to 3 weeks’ duration as the predominant symptom, which the clinician believes to be an infectious ALRTI, as defined in other studies [3]. The exclusion criteria will be a working diagnosis of a non-infective disorder, such as heart failure, pulmonary embolus, oesophageal reflux, or allergy; use of antibiotics in the previous 2 weeks; immunological deficiencies; and/or inability to provide informed consent or unable to follow the study procedures. Pneumonia and acute exacerbations of chronic obstructive pulmonary disease will not be deemed as exclusion criteria, because they are included in the ALRTI definition. The Standard Protocol Items: Recommendation for Interventional Trials (SPIRIT) Checklist is provided as Additional file 1.

### Sample size calculation

To calculate the sample size, we will use a type I error of 5% and a power of 80%, and we will assume that antibiotic prescribing will decrease by at least 15%, from 60% to 45%, in any one of the three intervention groups. We will also assume a drop-out rate of 12% and an intra-cluster correlation coefficient for antibiotic prescribing within practices of up to 0.07, based on two recent studies [14, 16]. We estimate that a sample of 788 (197 × 4) patients will be needed. Allowance for an inflation factor of 3.73 due to clustering and rounding of numbers for the four subgroups give an overall sample size of 2940 [17]. As a consequence, at least 147 patients will be recruited per centre.

### Setting and timing

General practices throughout Catalonia in the localities of the study centres (with different socioeconomic levels) will be approached, and at least three pairs of clinicians (GPs and nurses) in eligible centres will be invited to participate. Eligible centres will be stratified depending on the socioeconomic level and baseline antibiotic consumption, and only those that had not previously used any intervention to reduce antibiotic prescribing rates will be selected. They are expected to include 150 patients (75 patients in each autumn-winter season) in the trial.

### Trial interventions

**Fig. 1:**
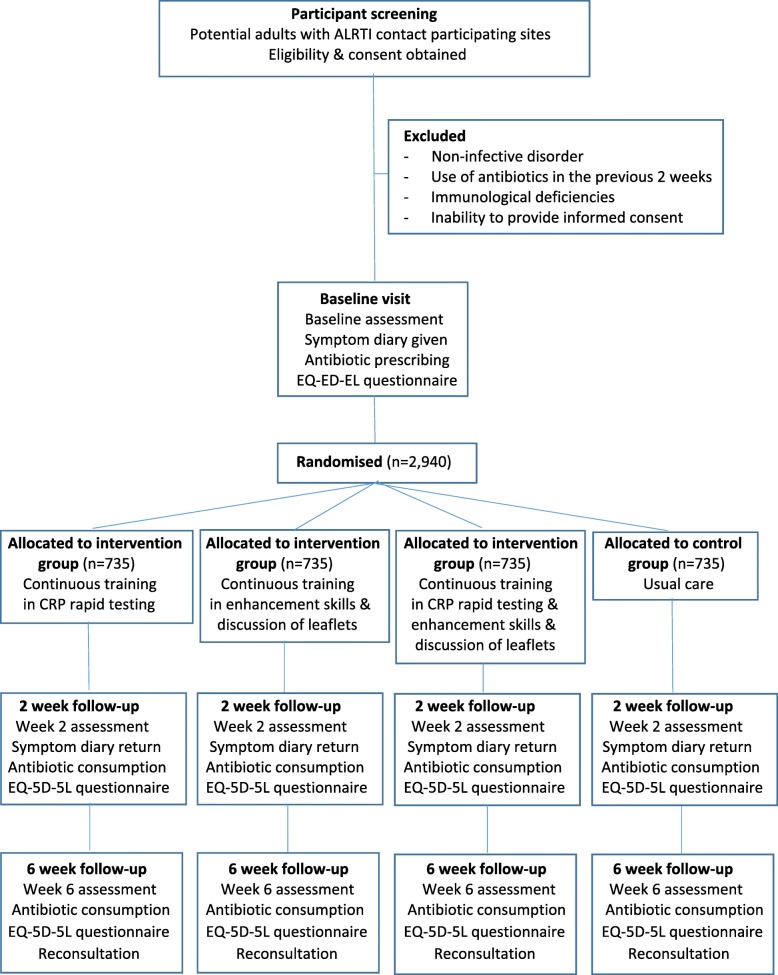
Flowchart of the randomised clinical trial. *ALRTI* acute lower respiratory tract infection

The centres will be randomly assigned to four trial arms: usual care, training in the use of a point-of-care CRP test, training in enhanced communication skills, or combined training in CRP testing and enhanced communication skills (Fig. 1). Masking of GPs or patients to the intervention itself is not possible. Randomisation of primary care centres will be achieved by computer generation of random numbers.

An on-the-spot 2-h training workshop will take place in the different areas before the inception of the trial. This training will be followed by monthly internet-based short training capsules tailored to either of the two interventions measured (clinical cases, medical literature, reminders) that GPs allocated to this intervention will have to attend and answer. GPs assigned to both interventions will receive the two training programmes. The usual care group will assess and manage patients according to the practice’s normal procedures.

The CRP group will receive internet training on how to target testing (e.g., in cases of clinical uncertainty, such as in patients with abnormal auscultation, dyspnoea, and impairment of vital signs) and how to negotiate with the patient about management decisions. The tests will be carried out with Afinion 2 CRP devices (Abbott Diagnostics, Lake Forest, IL, USA). Clinicians (GPs and nurses) will practise using the device during a run-in period of 2 weeks before data collection starts. GPs will be informed about the updated recommendations on how to interpret the CRP values and the evidence-based management (no antibiotics, delayed antibiotic prescribing or immediate antibiotic prescribing). Training in enhanced communication skills will focus on the gathering of information on patients’ concerns and expectations, exchange of information on symptoms, natural disease course and treatments, agreement of a management plan, red flags, and providing guidance about when to re-consult and summing up. GPs will also be provided with an interactive booklet to use during consultations that includes information on symptoms, use of antibiotics and antibiotic resistance, self-help measures, and when to re-consult, and the booklet will be given to the patient. This training will be supported by video demonstrations of consultation techniques. GPs will also be informed about the updated recommendations on management of ALRTIs.

### Outcome measures

During the index consultation GPs will document the duration of the illness, the severity of cough and other symptoms (rated 0, not problematic; to 6, severely problematic), and the severity of the infections. After randomisation a more detailed case report form (CRF) will be used in the follow-up consultations and will include the same details as the index form plus medical history, current medications, smoking status, findings of structured examination, whether CRP is tested, and whether the booklet is used. The primary outcome measures will be antibiotic consumption within the first 6 weeks, as documented in the CRFs and double-checked by the Pharmacy Unit of the Catalan Institute of Health, which can track if the antibiotic has been dispensed at any of the Catalan pharmacies; the duration of moderate to severe cough (i.e., those rating their cough as 3 or more by means of a Likert scale); and the quality-adjusted life-years (QALYs), which will be collected by means of the EQ-5D-5L questionnaire within the first 6 weeks (Table 1).

**Table 1:** Schedule of enrolment, interventions and assessments for the clinical trial

Time point	Study period November 4, 2019–April 30, 2021		
	Day 1	Week 2	Week 6
Enrolment			
Medical history and physical examination	X		
Explanation of the study and informed consent	X		
Initial CRF	X		
Giving out of the symptom diary, up to week 2	X		
Interventions			
CRP rapid test if needed (only among those patients visited by professionals assigned to the CRP intervention groups)*	X		
Use of an interactive booklet (only among those patients visited by professionals assigned to the enhanced communication skill intervention groups)*	X		
Assessments			
Assessment of the clinical outcome		X	X
Collection and review of the symptom diary		X	
EQ-5D-5L questionnaire	X	X	X
Antibiotic consumption	X	X	X
Use of drugs other than antibiotics	X	X	X
Tests ordered by clinicians	X	X	X
Patient satisfaction with care		X	X
Patient perception of the usefulness of the information received		X	X
Patient future consulting intentions		X	X
Absenteeism	X	X	X
Evaluation of adverse events		X	X
Number of re-consultations to primary and secondary care and number of complications regarding the ALRTI			X

*Abbreviations: ALRTI* acute lower respiratory tract infection, *CRF* case report form, *CRP* C-reactive protein, *GP* general practitioner

Randomisation will take place 2 weeks before initiating the clinical trial (October 21, 2019). Workshop for GPs and nurses assigned to any of the three intervention arms at the end of October 2019

*Monthly internet-based short training capsules for GPs and nurses assigned to any of the intervention groups

Secondary outcome measures are listed in Table 2. The duration of symptoms rated moderate to severe will also be recorded. Because this study will follow patients for 6 weeks, we will be able to better define the duration of acute cough due to ALRTI. Symptoms will be rated daily as 0 (no problem) to 6 (as bad as it could be) until they resolve, and the information will be reported by patients in self-completed diaries, similar to what was done in a recent clinical trial on delayed antibiotic prescribing carried out in Catalonia [18]. For patients who do not return diaries, a short form asking about the details of the duration and severity of symptoms and whether antibiotics have been taken or whether the booklet has been used will be completed by telephone call to the patient.

**Table 2:** Secondary objectives and outcome measures

Objectives	Outcome measures	Time point(s) of evaluation of this outcome measure
Number of re-consultations to primary and secondary care and number of complications regarding the ALRTI	Re-consultation for new or worsening symptoms, new signs, or hospital admission, assessed by review of medical notes (practice staff, the local study team, or both using a standard form to report these data), and number of complications regarding the ALRTI	First 6 weeks post-randomisation
Duration of moderate to severe symptoms	Number of days until the last day any of the symptoms is rated 3 or more. Symptoms will be rated daily as 0 (no problem) to 6 (as bad as it could be) until they resolve, and the information will be reported by patients in self-completed diaries.	First 6 weeks post-randomisation
Antibiotic prescription at the baseline visit, differentiating immediate and delayed antibiotic prescribing, and antibiotic dispensing at the pharmacies	Reported in the CRF. The number of patients treated with immediate and delayed antibiotic prescribing will be evaluated. Dispensing of the antibiotics at the pharmacies.	Baseline visit. The dispensing of antibiotics will be tracked in the first 6 weeks post-randomisation.
Drugs other than antibiotics	Reported by patients in self-completed diaries	First 6 weeks post-randomisation
Tests ordered by clinicians	Reported in the CRF	Baseline visit
Patient satisfaction with care	Reported in the symptom diaries	First 6 weeks post-randomisation
Patient perception of the usefulness of the information received	Collected in the symptom diaries	First 6 weeks post-randomisation
Patient future consulting intentions	Collected in the symptom diaries	First 6 weeks post-randomisation
Serious adverse events	Assessed by review of medical notes (practice staff, the local study team, or both using a standard form to report these data)	First 6 weeks post-randomisation
Number of days of sick leave (absenteeism)	Collected in the CRFs	First 6 weeks post-randomisation

*ALRTI* acute lower respiratory tract infection, *CRF* case report form

The data collection process and the rest of the study procedures will be supervised. A clinical research associate will perform periodic visits to the sites and provide online supervision of the data. A data monitoring committee has been deemed not necessary because of the study characteristics, as well as external audits. Nevertheless, the coordinating team will hold periodic meetings, and study procedures will be reviewed.

### Resource use and costs

Micro-costing techniques will be used to estimate the healthcare costs for patients with acute cough in the different interventions. The costs will be presented in 2021 euros (year foreseen to finalize the study). Primary data of the ISAAC-CAT clinical trial will be used whenever possible. The costs will include the costs of treatment to control the acute cough and management of these patients. The cost of treatment will depend on the state of health of the patient but may include the costs of the different therapies prescribed, including those recommended to be purchased at the pharmacies. The costs of management will include the cost of primary care and secondary care visits within the first 6 weeks as well as any diagnostic tests performed and the number of days of patient absenteeism. The cost of the interventions will cover the continuous training, including the workshop and the monthly delivery of training microcapsules, the cost of the booklets, the cost of the CRP machine, and the reagents used.

### Cost-effectiveness and cost-utility analyses

The cost-effectiveness analysis will be conducted comparing costs and effectiveness/utility, measured in natural units and QALYs, respectively, of the different alternatives to usual practice. In most cases the data will be obtained from the ISAAC-CAT clinical study. The comparison will be performed using the incremental cost-effectiveness ratio and the incremental cost-utility ratio, defined as the ratio of the difference in costs and the difference in effectiveness or utility. The results will be expressed in terms of euros per unit of effectiveness.

### Statistical analysis

Analyses will be carried out by intention to treat and will use multilevel logistic regression modelling with different distributions of errors according to the objective analysed for a factorial study controlled for the baseline antibiotic prescribing rate and with allowance for clustering by GP and health centre. The effects of various potential confounders related to clinical severity will be explored because of the potential for selection bias in an open trial. If interactions between interventions are not significant, the results will be presented as the main effects of each intervention (e.g., factorial groups with estimates controlling mutually for each intervention). The analysis will be performed using Stata 15 econometric software (StataCorp, College Station, TX, USA).

To study uncertainty, univariate and multivariate deterministic sensitivity analyses of the relevant parameters considered will be undertaken.

### Qualitative studies

There will be two qualitative studies, one pre- and one post-clinical trial. A phenomenologist framework will be applied in both studies. This is a relevant approach because the qualitative studies aim to explore the experiences of ALRTIs and healthcare in primary care settings among participants in their own words and through their own interpretations of their realities [19]. Sampling will be theoretical and purposive and will include a number of pathways to ensure maximum inclusivity.

#### Pre-clinical trial study

Semi-structured individual interviews (*n* = 30) and a group discussion (*n* = 8–12 participants) will be conducted with patients diagnosed with an ALRTI in the pre-clinical trial study. This qualitative pre-clinical trial study will aim to explore past experiences with ALRTIs, expectations and preferences of treatment, patients’ understanding of the symptoms of ALRTIs, the relationship with healthcare professionals, and perceived communication skills of healthcare professionals. Data collection will take place in the primary care clinics where participants are recruited. The interviews will take 45–60 min, and the group discussion will last about 90 min. They will all take place in the primary care centres. The semi-structured interviews and group discussion will be audio-recorded and transcribed systematically and verbatim. All data collection will be conducted by the same technical expert from the research team. The topic guide for the group discussion will be developed on the basis of findings from the interviews. All interviews will be conducted in the two official languages (Spanish and Catalan).

#### Post-clinical trial study

The aim of this study will be to investigate the satisfaction, pros, and cons of GPs, nurses, and patients assigned to the different intervention trial arms. For this study, 35 healthcare professionals (GPs and nurses) and 30 patients will be recruited. Recruitment will be carried out as for the pre-clinical trial study. The methods of data collection will be different for healthcare professionals and patients. Healthcare professionals will be asked to take part in a World Café [20], and patients will be recruited for individual semi-structured interviews as for the pre-intervention study. Through World Café we will generate GP and nurse collective knowledge about what are the preferences, needs, experiences, strengths and limitations in reducing inappropriate antibiotic prescribing for adults with ALRTIs.

#### Data analyses

A thematic content analysis will be performed on the basis of information obtained in the interviews and World Café. The data will be analysed in the following manner. After successive readings of the transcribed interviews, the researchers will attain some pre-analytical insight into the data. Next, four researchers will conduct the following analytical steps:

1. Identification of the relevant subjects and texts
2. Fragmentation of the text into units of meaning
3. Text codification with emerging codes from the data
4. Creation of categories by grouping the codes based on the criterion of similarity
5. Analysis of each category
6. Elaboration of new text with the results

These results will subsequently be discussed among the research team members until a consensus is reached (triangulation). The analysis will be performed using NVivo software (QSR International, Doncaster, Australia).

## Discussion

ALRTIs are very prevalent diseases and one of the most frequent causes of medical visits in primary care [21]. Despite most of these infections’ being self-limiting conditions, most patients feel ill, and many do not perform their usual activities. Patients often return to their physician or seek other medical help because the symptoms may persist, mainly cough, which may be very bothersome for some [22]. Furthermore, patients with bronchitis miss an average of 2–3 days of work per episode [23]. In addition, many patients are prescribed antibiotics unnecessarily.

Rapid testing, such as CRP testing, has been widely promoted for improving the care of with ALRTIs [1]. Most evaluations of diagnostic devices consider analytic performance without evaluating impact on patient outcomes or costs. However, new tests should not be introduced into routine clinical care if they do not improve outcomes that matter to patients individually or to society, including consideration of impact on recovery and quality of life, antibiotic prescribing, and antibiotic resistance [24]. Efficiency is also a relevant criterion for health decision making. The enhancement of clinician communication skills with the provision and discussion of leaflets targeted to the population has also demonstrated a powerful tool to reduce inappropriate antibiotic prescribing. However, the continuous training in these interventions has never been investigated.

### Trial status

Recruitment opened on 18 November 2019 and is expected to continue to 30 April 2021.

## Supplementary information

**Additional file 1.** Standard Protocol Items: Recommendations for Interventional Trials (SPIRIT) 2013 checklist: recommended items to address in a clinical trial protocol and related documents.

## Data Availability

The datasets generated and/or analysed during the current study are available in the Scientia repository (http://scientiasalut.gencat.cat).

## Abbreviations

ALRTI: Acute lower respiratory tract infection
CRF: Case report form
CRP: C-reactive protein
EQ-5D-5L: 5-level EuroQol 5 dimensions questionnaire
GP: General practitioner
ISAAC-CAT: Effectiveness of Improving Diagnostic and Communication Skills on Antibiotic Prescribing Appropriateness in Acute Cough
QALY: Quality-adjusted life-year
